# Dupuytren Disease: Is There Enough Comprehensive Patient Information on the Internet?

**DOI:** 10.2196/ijmr.7822

**Published:** 2017-06-22

**Authors:** Grzegorz Zuk, Katharina B Reinisch, Dimitri A Raptis, Sonia Fertsch, Merlin Guggenheim, Adrian F Palma

**Affiliations:** ^1^ Hospital of Wetzikon Department of Surgery Wetzikon Switzerland; ^2^ Cantonal Hospital of Olten Department of Surgery Olten Switzerland; ^3^ Sana Hospital Department of Plastic and Esthetic Surgery Düsseldorf Germany; ^4^ Department of Plastic Surgery and Hand Surgery University Hospital Zürich Zürich Switzerland

**Keywords:** congenital hand deformity, Internet, patient education

## Abstract

**Background:**

Dupuytren disease is a chronic nonmalign fibroproliferative disorder that causes finger contractures via proliferation of new tissue under the glabrous skin of the hand, resulting in multiple functional limitations for the patient. As many surgical therapy options exist, patients suffering from this condition actively search for information in their environment before consulting a health professional.

**Objective:**

As little is known about the quality of Web-based patient information, the aim of this study was to conduct its systematic evaluation using a validated tool.

**Methods:**

A total of 118 websites were included, and qualitative and quantitative assessment was performed using the modified Ensuring Quality Information for Patients (EQIP) tool. This standardized and reproducible tool consists of 36 items to assess available information in three categories: contents, identification, and structure data. Scientific data with restricted access, duplicates, and irrelevant websites were not included.

**Results:**

Only 32 websites addressed more than 19 items, and the scores did not significantly differ among the website developers. The median number of items from the EQIP tool was 16, with the top websites addressing 28 out of 36 items. The quality of the newly developed websites did not increase with passing time.

**Conclusions:**

This study revealed several shortcomings in the quality of Web-based information available for patients suffering from Dupuytren disease. In the world of continuously growing and instantly available Web-based information, it is the health providers’ negligence of the last two decades that there are very few good quality, informative, and educative websites that could be recommended to patients.

## Introduction

Dupuytren disease was named after a French surgeon who first described and operated on it in the early thirties of the 19th century [1]. It is a chronic nonmalign fibroproliferative disorder that causes finger contractures by affecting the palmar aponeurosis of the hand. For the patient, it is associated with multiple functional limitations of the hand [2]. Usually the metacarpophalangeal- (MCP) and proximal interphalangeal- (PIP) joints in the fingers are involved [3]. Less frequently, Dupuytren disease affects other parts of the body, for example, soles of the feet and penis [4]. Typically it consists in progressive formation of fibrous nodules and cords, leading finally to a flexion contracture. As this condition is quite common, reaching an overall incidence of approximately 5% and 20% at the age of over 65 years [5], there are lots of patients actively searching the Internet for possible therapy options and for aids in decision making before consultation with a health professional [6]. Therefore, comprehensive and easily available patient information is an issue of great interest in community health. The Internet is a constantly growing medium containing all kinds of information instantly available for every user and medical information is no exception. However, the Internet is also an uncontrolled space without any guarantee of the correctness of the information presented. Thus, a website developer is solely responsible to provide accurate, professional, and objective medical information [7]. The International Patient Decision Aid Standards (IPDAS) collaboration has established international guidelines for the development of health care decision aids using the Web-based Delphi consensus process [8,9], which were revised in 2013 and converted to a checklist consisting of 44 items [10]. Another validated instrument to assess the quality of patient decision aids is the Ensuring Quality Information for Patients (EQIP) instrument [11]. This tool, in the form of a checklist, was further expanded to meet the IPDAS criteria and the guidelines of patient information appraisal of the British Medical Association [12]. The EQIP instrument has successfully been used by many authors [13-18].

The aim of this study was to systematically evaluate the available Web-based information for patients with Dupuytren disease. The few existing papers on this topic report the quality of the available patient information to be poor [19,20]. To the best of our knowledge, an assessment of such information using a validated tool has never been done.

## Methods

### Eligibility, Information Sources, and Website Selection

Different combinations of the key words “Dupuytren’s contracture,” “Dupuytren’s surgery,” and “Dupuytren’s therapy” were used to identify websites in English only by 5 most popular [21] search engines: Google, Bing, Yahoo, Ask, and AOL. The geographic option in the search engines was switched off to avoid selection bias. For further assessment, we selected the first 100 search results for each search engine based on the assumption that Internet users limit their search to a number far below 100 [15]. To the selected 500 websites, the following noninclusion criteria were applied: websites not specific for Dupuytren disease, those related to articles in scientific journals, duplicates, and websites in language other than English. This resulted in the selection of 118 websites for further assessment.

### Patient Information Evaluation Instrument

To assess each website, we used the modified EQIP tool [12], which is a checklist consisting of 36 items and evaluates data in three different categories: (1) content data, (2) identification data, and (3) structure data (Table 1).

The EQIP tool was developed by rating the quality of 73 documents describing medical care procedures used at the University Hospital of Geneva, Switzerland. The assessment rules were defined on 25 documents, and two assessors independently rated the remaining 48 documents. The interrater reliability was very good (kappa statistic=.84), and the intraclass correlation coefficient was as high as .95 [16]. Although the EQIP tool included a 4-option rating scale of “yes,” “partly yes,” “no,” and “NA” (not applicable) in its native form, we decided to use its modified version with a binary scale of “yes” versus “no” or “NA” (ie, no score) after Melloul et al [15]. This is because the answer “partly yes” is, in our opinion, too subjective. Furthermore, there is evidence that associates this answer with low dependability in the assessment of website content [22].

### Data Assessment

The data were independently assessed by three investigators and divergent results were defined by consensus. The obtained data were entered into a Web-based platform built on the open source content management system Drupal (version 7) [23], which guaranteed a standardized and complete data entry. According to the origin of the information, the 118 websites were categorized into 8 groups: (1) academic center, (2) encyclopedia, (3) hospital, (4) industry, (5) news service (the press), (6) practitioner, (7) professional society, and (8) patient group. Another classification was performed regarding the country of origin of the websites: (1) Australia, (2) Azerbaijan, (3) Canada, (4) France, (5) Germany, (6) New Zealand, (7) Singapore, (8) United Kingdom, and (9) United States.

### Morbidity Risks Associated With Surgical Treatment of Dupuytren Disease

To assess these risks, items 9 and 10 were applied (Table 1). Item 9 evaluates the description of qualitative risks and side effects or complications of surgical interventions (eg, if the risk of postoperative complications is mentioned on the website). Item 10 assesses the description of the quantitative risks of surgical techniques.

### Statistical Methods

Proportions derived from nominal variables were compared with Fisher or chi-square tests and continuous variables were compared with the Student *t* test or 1-way analysis of variance (ANOVA) test, where applicable. All *P* values were 2-sided and considered statistically significant when *P*<.05. According to the 36 items of the expanded EQIP tool, all 118 websites were scored from 0 to 36. Each item was given equal weight of importance. The 75th quartile was arbitrarily used as a cut-off point to differentiate high-score websites from low-score ones, and we dichotomized the obtained EQIP score as previously performed by Melloul [15]. Statistical analysis was performed with SPSS version 22 for Mac (IBM Corp).

**Table 1:** Overall results of the included websites according to the modified Ensuring Quality Information for Patients (EQIP) Instrument.

Data	Item	Criteria	Yes, n (%)	No, n (%)	Does not apply, n (%)
**Content data**					
	1	Initial definition of which subjects will be covered	43 (36.4)	75 (63.6)	0 (0)
	2	Coverage of the previously defined subjects (NA^a^if the answer is “no” for item 1)	43 (36.4)	75 (63.6)	0 (0)
	3	Description of the medical problem	115 (97.5)	3 (2.5)	0 (0)
	4	Definition of the purpose of the surgical intervention	103 (87.3)	15 (12.7)	0 (0)
	5	Description of treatment alternatives	86 (72.9)	32 (27.1)	0 (0)
	6	Description of the sequence of the surgical procedure	59 (50.0)	59 (50.0)	0 (0)
	7	Description of the qualitative benefits to the recipient	58 (49.2)	60 (50.8)	0 (0)
	8	Description of the quantitative benefits to the recipient	11 (9.3)	107 (90.7)	0 (0)
	9	Description of the qualitative risks and side effects	66 (55.9)	52 (44.1)	0 (0)
	10	Description of the quantitative risks and side effects	23 (19.5)	95 (80.5)	0 (0)
	11	Addressing quality-of-life issues	64 (54.2)	54 (45.8)	0 (0)
	12	Description of how complications are handled	10 (8.5)	108 (91.5)	0 (0)
	13	Description of the precautions that the patient may take	25 (21.2)	93 (78.8)	0 (0)
	14	Mention of alert signs that the patient may detect	20 (16.9)	98 (83.1)	0 (0)
	15	Addressing medical intervention costs and insurance issues	9 (7.6)	109 (92.4)	0 (0)
	16	Specific contact details for hospital services	48 (40.7)	70 (59.3)	0 (0)
	17	Specific details of other sources of reliable information or support	47 (39.8)	71 (60.2)	0 (0)
	18	Coverage of all relevant issues for the topic (summary item for all content criteria)	0 (0)	118 (100)	0 (0)
**Identification data**					
	19	Date of issue or revision	52 (44.1)	66 (55.9)	0 (0)
	20	Logo of the issuing body	111 (94.1)	7 (5.9)	0 (0)
	21	Names of the persons or entities that produced the document	37 (31.4)	81 (68.6)	0 (0)
	22	Names of the persons or entities that financed the document	1 (0.8)	117 (99.2)	0 (0)
	23	Short bibliography of the evidence-based data used in the document	37 (31.4)	81 (68.6)	0 (0)
	24	Statement about whether and how patients were involved or consulted in the document’s production	51 (43.2)	67 (56.8)	0 (0)
**Structure data**					
	25	Use of everyday language and explanation of complex words or jargon	111 (94.1)	7 (5.9)	0 (0)
	26	Use of generic names for all medications or products (NA if no medications described)	35 (29.7)	83 (70.3)	0 (0)
	27	Use of short sentences (<15 words on average)	109 (92.4)	9 (7.6)	0 (0)
	28	Personal address to the reader	33 (28.0)	85 (72.0)	0 (0)
	29	Respectful tone	118 (100)	0 (0)	0 (0)
	30	Clear information (no ambiguities or contradictions)	116 (98.3)	2 (1.7)	0 (0)
	31	Balanced information on risks and benefits	16 (13.6)	102 (86.4)	0 (0)
	32	Presentation of information in a logical order	115 (97.5)	3 (2.5)	0 (0)
	33	Satisfactory design and layout (excluding figures or graphs)	91 (77.1)	27 (22.9)	0 (0)
	34	Clear and relevant figures or graphs (NA if absent)	21 (17.8)	97 (82.2)	0 (0)
	35	Inclusion of a named space for the reader’s note or questions	3 (2.5)	115 (97.5)	0 (0)
	36	Inclusion of a printed consent form contrary to recommendations (NA if not from hospitals)	2 (1.7)	116 (98.3)	0 (0)

^a^NA: not applicable.

## Results

### Websites Containing Information on Dupuytren Disease

After screening 500 eligible websites, 118 websites were included for qualitative and quantitative analysis with the expanded EQIP tool. The criteria for noninclusion were duplicates and noneligible Web contents.

### Country of Origin and Source of Patient Information

**Figure 1:**
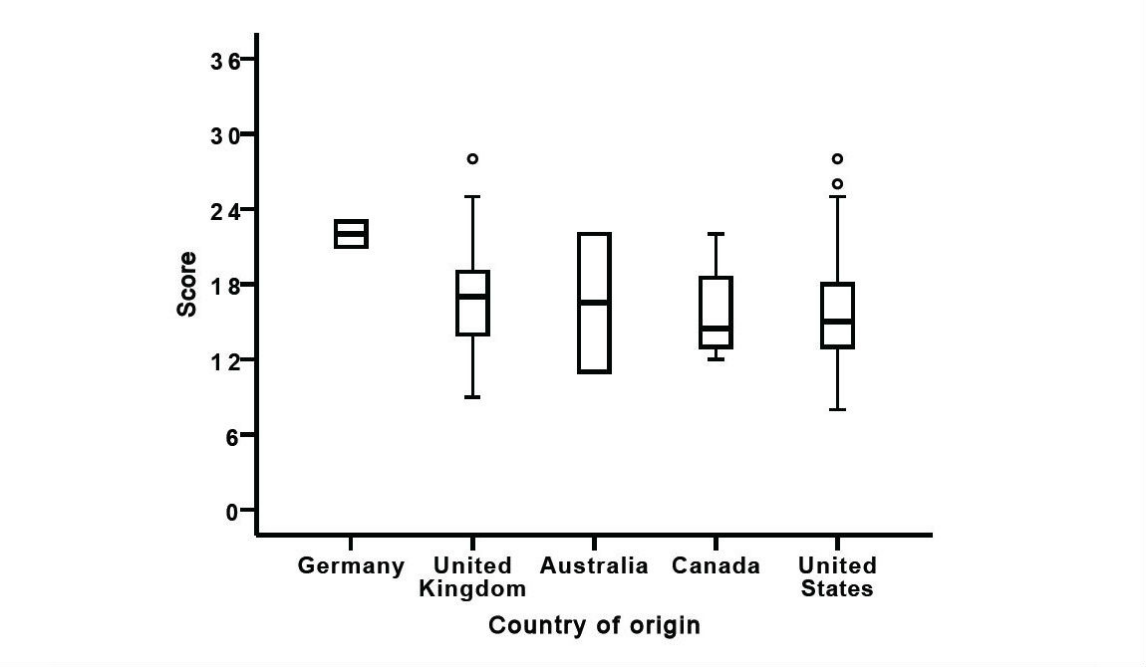
Box plot presenting website scoring based on the modified Ensuring Quality Information for Patients (EQIP) tool depending on country of origin. The horizontal thick line within the box plot represents the median. The upper line of the box plot represents the 75th percentile, whereas the lower the 25th percentile. The upper whisker line represents the maximum value, whereas the lower the minimum value. Outliers are shown as circles.

More than two-thirds (75.4%, 88/118) of all websites originated from the United States, followed by the United Kingdom (14.4%, 16/118). Canada was represented in 3.4% (4/118). Additionally, 23.6% (21/89) of the 89 US websites were rated as high-score websites, which made 65.6% of all (n=32) high-score websites (Figure 1).

**Figure 2:**
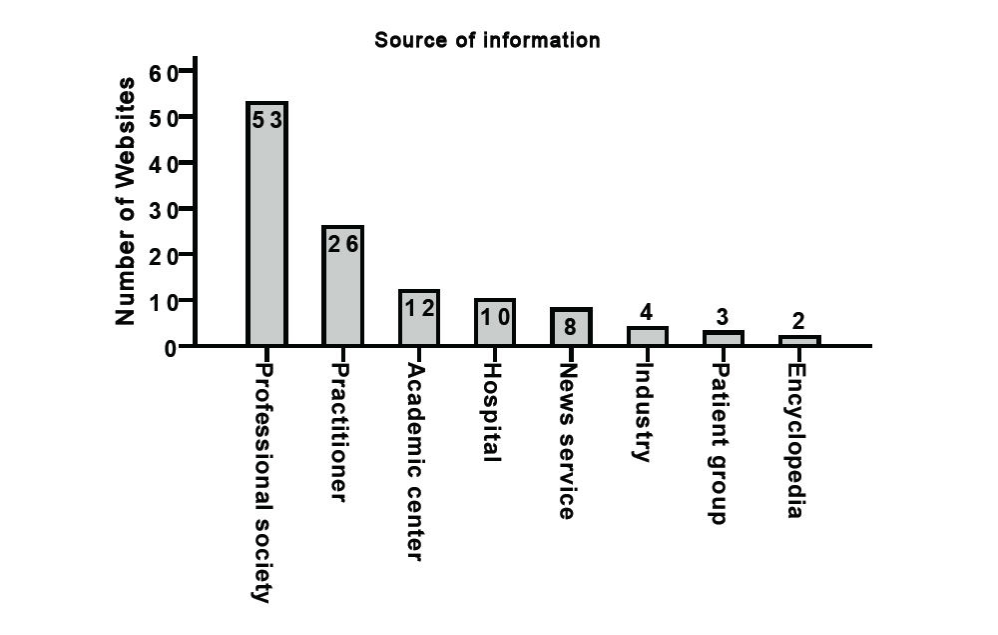
Distribution of the total 118 evaluated websites depending on source of information.

Fifty-three websites (44.9%, 53/118) were developed by professional societies, which thus represent the most frequent source of information on Dupuytren disease. Practitioners were the source number 2 with 26 websites (22%, 26/118; Figure 2 **)**.

### Ensuring Quality Information for Patients (EQIP) Score Achieved

**Figure 3:**
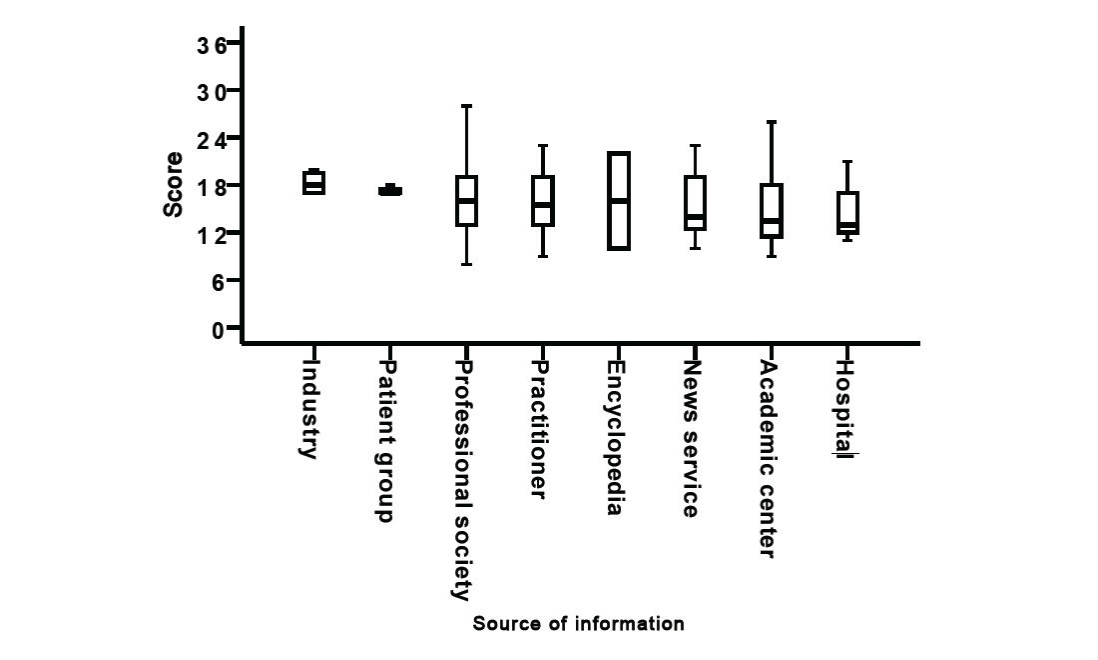
Box plot presenting website scoring based on the modified Ensuring Quality Information for Patients (EQIP) tool depending on source of information.

The median website score obtained from the EQIP tool was 16 points (interquartile range, IQR: 13-19). The lowest score of 8 points was achieved by one website and the highest score of 28 points by two websites. None of the screened websites provided information on all 36 items of the modified EQIP tool. When the source of medical patient information was concerned, there was no statistically significant difference between scores obtained by different website developers (Figure 3).

**Figure 4:**
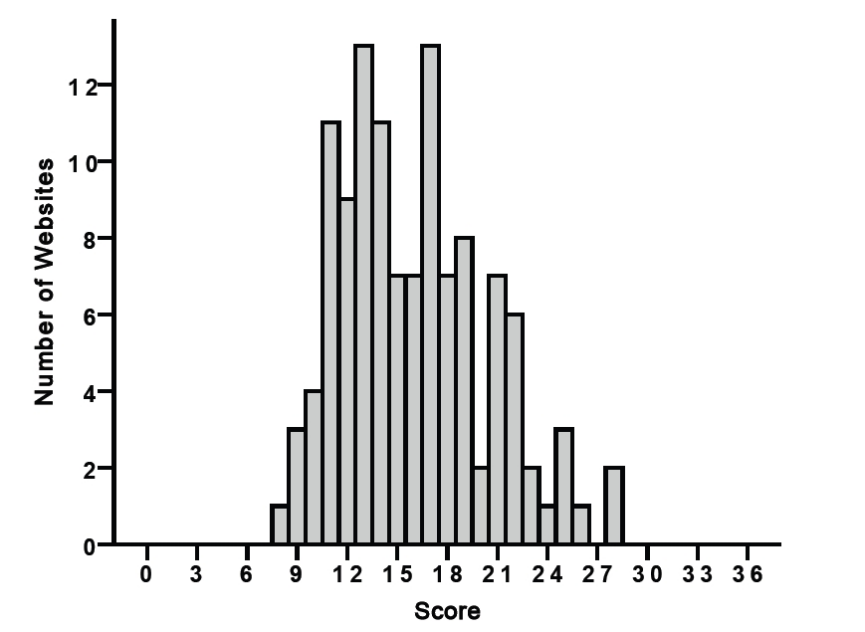
Histogram presenting the number of websites (Y=vertical axis) and their scores according to the modified Ensuring Quality Information for Patients (EQIP) instrument (X=horizontal axis).

Websites above the 75th percentile (with the score of 19 or more) were defined as high-score websites, in contrast to low-score websites (obtaining 18 points or less). A high score was achieved by 32 websites (27.1%, 32/118) and a low score by 86 websites (72.9%, 86/118) **(**Figure 4 **).**

### Top Rated Websites

We defined a top rated website with a score above the 95th percentile (Table 2 **)**. The top rated websites came from the United Kingdom (n=2) and from the United States (n=4). The highest score reported was 28, ex aequo from a British professional society and from an American professional society.

**Table 2:** The top rated websites (>95th percentile) according to the modified Ensuring Quality Information for Patients (EQIP).

Ranking	Website	Country of origin	Source of information	Score
1	http://dupuytrens-society.org/index.html	United Kingdom	Professional society	28
1	http://www.cig0.com/healthwellness/hw/medical-topics/dupuytrens-disease-ue4602	United States	Professional society	28
2	http://depts.washington.edu/uwhand/Therapy/dupuytrens.php	United States	Academic center	26
3	http://www.emedicinehealth.com/dupuytrens_disease-health/article_em.htm	United States	Professional society	25
3	http://www.nhs.uk/conditions/Dupuytrens-contracture/Pages/Introduction.aspx	United Kingdom	Professional society	25
3	http://www.orthop.washington.edu/?q=patient-care/hand/dupuytrens-disease.html-0	United States	Academic center	25

**Figure 5:**
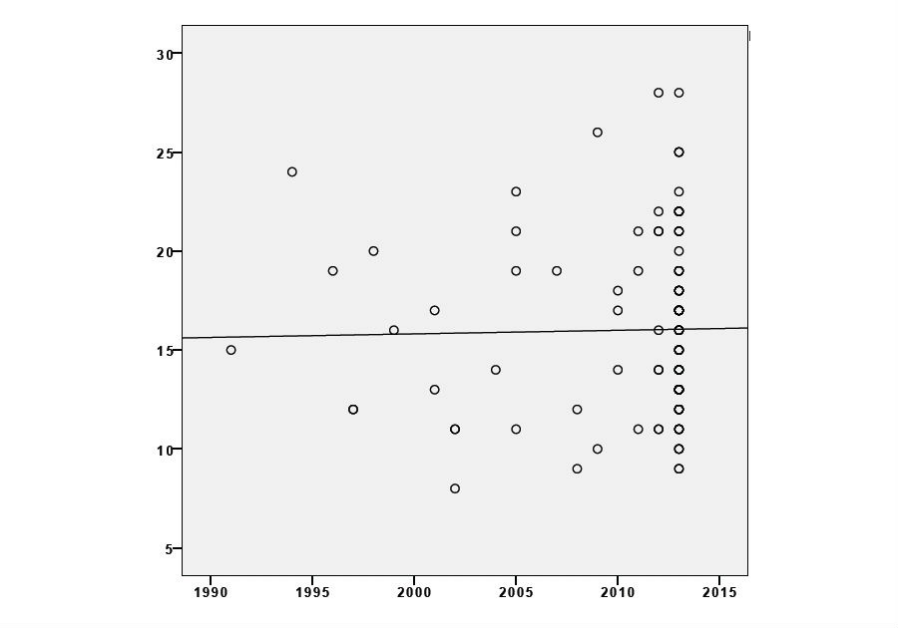
Scatter plot with the year of website publication on the horizontal axis (X) and their scores awarded by the modified Ensuring Quality Information for Patients (EQIP) instrument on the vertical axis (Y). The solid line represents the mean EQIP score of the websites.

### Year of Publication

More than two-thirds (68.6%, 81/118) of the websites screened were published in 2013 in contrast to 37 websites published from 1990 to 2012. Within the passing time, the EQIP-based quality of the newly introduced websites did not increase significantly, as shown in Figure 5.

## Discussion

### Principal Findings

The most important findings of the study were, first, that the overall quality of patient information on Dupuytren disease evaluated with a validated tool was poor. Second, the source of medical patient information did not influence the scores obtained by the websites. Third, none of the screened websites provided information on all 36 items of the modified EQIP tool, and the high-score websites represented only a quarter of the screened websites. Finally, the quality of the newly developed websites did not increase with passing time.

The Internet presents a global, easily accessible, and unlimited source of any kind of information, and medical issues is one of the most searched topics. It is also an uncontrolled space, allowing anyone to put any kind of information out there, and also that of unknown accuracy. This may expose patients to the risk of getting wrong information and impact their further therapeutic decisions. These concerns led various authors to investigate the accuracy of the medical information for patients in different medical disciplines. [15,16,24-26]

The systematic evaluation of the quality of Internet information on Dupuytren disease is sporadically present in the literature in contrast to the information on other common hand pathologies.

Sproule et al [19] conducted in 2003 an evaluation of 172 websites containing medical information on 3 common hand pathologies such as Dupuytren disease, carpal tunnel syndrome, and trigger finger. The published patient information was evaluated for completeness and accuracy using a scoring system developed by the authors. The findings of that study in terms of those two evaluation criteria showed substantial shortcomings in most websites. In contrast to the methodology of our study, Sproule et al did not use a validated evaluation scoring system.

Almost a decade later, Kelly et al [20] performed an Internet search of “Dupuytren’s disease” using the most popular search engines. The identified websites were scored using the DISCERN scoring system [27] and the Journal of American Medical Association (JAMA) benchmark criteria [28]. Compared with other common hand pathologies examined in that study, the quality of the Internet information on Dupuytren disease measured by DISCERN and JAMA criteria was better, but nevertheless, the study revealed a small number of websites that could be recommended to patients to support their decision making in the therapeutic process. The used evaluation tool—the DISCERN instrument—was developed by an expert panel and comprises 16 criteria for judging the quality of written consumer health information on treatment choices. Although the instrument requires some subjectivity for rating certain criteria, its developers claim it to be reliable and valid [27], and this could be verified by other authors. [29-31] In contrast to the EQIP instrument, the DISCERN evaluates information on treatment choices but does not evaluate readability or design aspects of the written materials. In our opinion, the EQIP is a more comprehensive and practical tool to evaluate the large, constantly growing volume of patient information produced within the health service. It helps also to make decisions about the urgency of any revisions that are needed to be made to written information in order to prioritize limited resources and minimize costs [11].

This study shows that private institutions did not provide less quality of information in comparison with academic nonprofit oriented website developers. Since the market of hand surgery, especially in the private setting, is consumer-oriented and strongly relies on marketing and advertising tools in an increasing crowded field of providers, physicians tend to advertise their services with complete patient information. This tempts the physician to take marketing action of selling his “products” and to influence the patient’s interest. However, economic issues should never yield to medical responsibilities and ethics.

### Limitations

This study has some limitations. First, due to the assumption that English is spoken as the first or second language in most developed countries, only websites developed in English were included; therefore, the quality of websites published in other languages remains unknown. The same can refer to the selection of search engines. Second, this work was done according to the statistical popularity of the search engines [21]; nevertheless, the use of other search engines could have revealed other interesting websites. Third, the Internet is a highly dynamic and constantly growing medium, and an evaluation of 118 websites at one point of time can represent only a snapshot of the information provided on the Web. Finally, there were limitations in the assessment instrument itself. The modified EQIP tool and its scoring system was not designed to assess websites referring specifically to Dupuytren disease but rather to assess patient information regarding any kind of medical treatment, which could have led to interpretation bias.

### Conclusions

The evaluation of the present Web-based patient information on Dupuytren disease using a validated tool revealed substantial shortcomings and lacked standardization of its quality. The health care providers are the first to blame for this condition because in their obligation to provide a patient with an accurate and complete information, they did not stay up to date and recognize the potentials and hazards of this continuously growing medium—the Internet.

## Abbreviations

EQIP: Ensuring Quality Information for Patients
IPDAS: International Patient Decision Aid Standards
IQR: interquartile range
